# New York Odontological Society

**Published:** 1885-11

**Authors:** 


### NEW YORK ODONTOLOGICAL SOCIETY.

Reported for the Independent Practitioner.

Arrangements having been completed for the meetings of this Society to be permanently held in the parlors of the New York Academy of Medicine, No. 12 West 31st Street, the first gathering of the members in their new quarters took place on Tuesday evening, October 13th.

The President, Dr. William Jarvie, read an address, giving a brief history of the Society from its inception to the present time, what it had accomplished in the past and its possibilities for future usefulness, stating that up to the present time the meetings of the Society had been held at the residences of individual members, which had given opportunities to visit the operating rooms of many prominent members of our calling, where ample light had been afforded for examining office appointments, appliances, instruments, etc., and for witnessing cases of peculiar operation. All this, together with the social element, which had been engendered by their former mode of coming together, had tended to make the occasions of their meetings exceedingly interesting and enjoyable. But the time had come when a change was deemed advisable, and all the members can now have equal opportunities of doing each their share in the way of contributing to the welfare of the Society. It is the intention of the Society to have a library and museum, to which all the members and friends are invited to contribute books, appliances, mechanical devices, instruments, and specimens of interest of every type suitable to the purpose, that can be procured.

At the close of his address President Jarvie introduced Prof. Abraham Jacobi, M. D., President of the Academy of Medicine, who, in behalf of the members of the Academy, gave an address of hearty welcome to the Odontological Society to their new place of meeting. Dr. Jacobi, in the course of his remarks, dwelt at length upon the relations of modern dentistry to general medicine, and considered it one of the branches of the great healing art, contributing largely in the way of ministering to human ills, of alleviating the physical sufferings of mankind, and doing its part in the way of insuring health and comfort to the race. The careful study of embryology, he claimed, divulged the fact that many of the mortal ills had their origin in the earliest stage of fœtal formation, and that various taints of parental weakness were stamped on the embryonic germ which is to develop and become an animated human being.

The peculiar characteristics in the formation of teeth, respecting shape, color, texture, etc., were referred to as types of organic structure, showing conditions of health and marks of early disease. He referred also to the great advances made in dental science, which had followed in the train of dental art, and believes that dentistry is to play an exceedingly important part as a specialty of medicine.

The excellent address of Dr. Jacobi was listened to with profound attention and interest.

Dr. J. Smith Dodge, Jr., responding for the Odontological Society, alluded in fitting terms to the warm words of welcome embodied in the address of Dr. Jacobi, the sentiments of which were greatly appreciated. He spoke also of the mission of the modern dentist, and the strange yet somewhat prevailing misconception of their duties and requirements, and that, although there were some men who call themselves dentists and who occupy a very limited sphere in the field of dental labor, the earnest and prominent members of the profession are constantly reaching forward, grasping at whatever science may reveal that bears upon their special duties, and giving much time in the way of research and investigation, in order to fit themselves more thoroughly and perfectly for the labors of their calling.

President Jarvie introduced Dr. John A. Wyeth, stating that he had several patients in an adjoining room, for whom he had operated, and whom he would bring before the Society in order that the results of his operations might be witnessed by the gentlemen present.

The first patient showed a staphyloraphic operation which, viewed from a surgical standpoint, was a decided success, showing a marked degree of skill on the part of the surgeon. The operation was rendered painless by a free application of hydrochlorate of cocaine.

Another patient was brought forward showing a case of diseased antrum, which was still under treatment. From the angle of the mouth to a point some three inches back, the cheek had been laid open to secure access to the diseased territory. A case of alveolar abscess was also presented, which still discharged through an opening in the cheek. A fourth case was that of a lad apparently ten years of age, for whom the surgeon had removed the left half of the inferior maxilla in order to dispose of a malignant sarcoma.

*Dr. J. Morgan Howe*—Stated that the operation performed by Dr. Wyeth for cleft palate was a beautiful operation, but he desired to know what indications would warrant such operations, and what benefit to the patient would be gained thereby.

In reply, Dr. Wyeth stated that it was an aid to deglutition, and prevented the food from being forced into the pharynx and nares when taking nourishment. He thought also that it somewhat improved the speech, but if this latter object was to be considered the operation should be done quite early in life, as it is difficult to break up a long acquired habit of articulating words, or to unlearn what had become thoroughly learned.

*Prof. Frank Abbott*—Asked Dr. Wyeth if he deemed it better to operate in such cases than to furnish artificial appliances.

*Dr. Wyeth*—Was not so positive as regards an improvement in speech, yet he believed it a good thing to operate, and as he possessed a fine set of instruments, devised by Dr. Goodwillie, he should continue to operate so long as he could find subjects to work upon.

*Dr. N. W. Kingsley*—Regretted the last remark of the gentleman who preceded him. He did not believe that Dr. Wyeth or any other surgeon, if open to reason and conviction, would ever operate for cleft palate if they had seen such results as he had witnessed from surgical operations, and from artificial appliances. The latter will render speech perfect, but surgery is a failure. The contracted condition of the velum, resulting from the surgical union, makes perfect articulation impossible. Where the palate is defective, speech is always defective, consequently surgical operations never improve speech. He declared surgical operations of this sort worse than useless, for where performed they prevent the use of artificial appliances, and were no gain in any respect. "Art," said the speaker, "gives the means of benefit, but surgery does not."

As regards deglutition, Dr. Kingsley did not believe that it was benefited in the least by surgery. He had carefully studied this matter, and in the many cases that had come to his notice no marked improvement was ever made in this respect by surgical operations.

As the hour of adjournment was reached, the discussion of other matters was postponed, and the members were invited to a sumptuous repast that had been prepared, where all could test their own powers of deglutition.

